# Supplementary material for: High-resolution kinetic characterization of the RIG-I-signaling pathway and the antiviral response
Source: Life Sci Alliance. 2023 Aug 9;6(10):e202302059. doi: 10.26508/lsa.202302059 (PMC10412806; doi:10.26508/lsa.202302059)
Supplement: Supplementary file 1 [file LSA-2023-02059_Supplemental_Data_1.docx]

# Supplement: A mathematical model of the antiviral innate immune response

## 1.1 Data normalization

To compare western blot intensities between different conditions and to combine multiple biological replicates, raw data were normalized. It was not possible to quantitatively compare intensity values of non-identical western blot gels, but only intensities, which were measured on the same gel. This is due to differences in antibody binding and slightly varying settings during the exposure. Consequently, it is important to consider this prior to conducting the experiments and include controls to be able to combine western blot intensities.

Figure 1.1: A) Observed dynamics of RIGI pathway components after stimulation with double-stranded RNA using electroporation. Phosphorylated and total protein levels were measured over time by western blotting and are in good agreement between biological replicates. Intensities are normalized as described in Section [1.1](#dataNormRIGI). Blue points correspond to data measured in a short time-period experiment (up to 4 h.p.e.) and yellow points correspond to data obtained from long time-period experiments (up to 24 h.p.e.). B) Normalized intensity values for every time-point that is measured over both experimental time-periods (short time-period experiments, long time-period-experiments) as mean and standard deviation. The proximity to the line of unity (dashed line) indicates little differences in the normalized intensity values for the different experimental protocols.

For the dsRNA transfection experiments, all protein intensities are normalised replicate-wise with respect to the mean of the 0 h, 1 h, 2 h, 4 h intensities of a protein. The time-points are measured in all experiments, i.e. short time-period experiments (up to 4 h.p.e.) and long time-period experiments (up to 24 h.p.e.). The resulting normalized intensities of RIGI pathway components are shown in Figure [1.1](#FigExpData) A for the first 4 h.p.e. The figure further indicates whether normalized intensities are measured in short time-period (yellow) or long time-period experiments (blue). We observe similar dynamics and normalized intensity values in the replicates and experimental setups, demonstrating the robustness of our normalization method. To further reveal potential errors introduced by our normalization method, we additionally compare the normalized intensity values of all time-points measured over both time-period against each other (Figure [1.1](#FigExpData) B). The normalized intensities are close to the line of unity (dashed line) for nearly all species and time-points, which indicates that we can quantitatively compare normalized intensity values irrespective of the time-period of the experiment. Only normalized IkB values at time-point 0 h.p.e. are markedly increased in the short time-period experiments compared to the long time-period experiments.

## 1.2 Model development: A RIG-I pathway model

Using the set of time-resolved western blot data and IFN qPCR data, we develop an ODE-based mathematical model of the early RIGI pathway dynamics after stimulation with double-stranded RNA, a mimic of viral replication intermediates. The model is limited to pathway components for which experimental data are present, with the single exceptions being RIGI and MAVS, which activation dynamics is experimentally hard to assess. The proposed model includes two compartments, namely the cytoplasm and the nucleus, compromising a total number of 19 species. It contains 20 rate constants as well as additional parameters to account for experimental conditions and the semiquantitative nature of western blot data (see Section [1.2.2](#SecParamEstCore)). In the following, we introduce the biological processes that are considered in the model and the resulting ODEs before addressing the parameter estimation.

### 1.2.1 Model description

A simplified representation of the model is shown in Figure [1.2](#coreModelOverview). The stimulation of the RIGI pathway is achieved by electroporation of dsRNA into A549 cells. This short-lived process is not explicitly included in our model but accounted for by assuming an equal concentration of dsRNA in both extracellular space and the cytoplasm at the start of the experiment as a consequence of the electropermeabilization. Cytosolic dsRNA consequently undergoes sensing and binding by RIGI proteins with rate constant $k_{\mathrm{RIGI}}$ or is degraded by the cellular RNA degradation system with $\mu_{\mathrm{dsRNA}}$. Formed RIGI/dsRNA complexes (aRIGI) interact with MAVS and produce active MAVS comples (aMAVS) with the rate constant $k_{\mathrm{MAVS}}$. RIGI/dsRNA complexes can further dissociate and activated MAVS complexes can be inactivated (rate constants $b_{\mathrm{RIGI}}$ and $b_{\mathrm{MAVS}}$, respectively). Including deactivation processes enables the simulation of long-lasting or repeated stimulation scenarios and can thus produce insights into cellular adaption. In addition, a baseline protein synthesis ($k_{syn,RIGI}$) and degradation ($\mu_{\mathrm{RIGI}}$) of RIGI is included in our model as it allows to simulate consequences of IFN-induced RIGI upregulation on the system. The early steps of the RIGI pathway are represented by the following set of ODEs:

$$\begin{matrix} \frac{d}{dt}dsRNA= & -k_{\mathrm{RIGI}}\cdot dsRNA\cdot RIGI+b_{\mathrm{RIGI}}\cdot aRIGI-\mu_{\mathrm{dsRNA}}\cdot dsRNA, \\ \frac{d}{dt}RIGI= & -k_{\mathrm{RIGI}}\cdot dsRNA\cdot RIGI+b_{\mathrm{RIGI}}\cdot aRIGI+k_{syn,RIGI}-\mu_{\mathrm{RIGI}}\cdot RIGI, \\ \frac{d}{dt}aRIGI= & +k_{\mathrm{RIGI}}\cdot dsRNA\cdot RIGI-b_{\mathrm{RIGI}}\cdot aRIGI-\mu_{\mathrm{RIGI}}\cdot aRIGI, \\ \frac{d}{dt}MAVS= & -k_{\mathrm{MAVS}}\cdot MAVS\cdot aRIGI+b_{\mathrm{MAVS}}\cdot aMAVS, \\ \frac{d}{dt}aMAVS= & +k_{\mathrm{MAVS}}\cdot MAVS\cdot aRIGI-b_{\mathrm{MAVS}}\cdot aMAVS. \end{matrix}$$

Activated MAVS (aMAVS) recruit a variety of proteins, among them kinases that trigger the activation of transcription factors IRF3 and NF-kappaB. In case of IRF3, MAVS complexes trigger the phosphorylation of TBK1 and IKKe (pTBK1, pIKK$\epsilon$). Due to the high level of homology ($\approx64\%$, Pomerantz 1999) between TBK1 and IKKe and proposed redundant roles as serine kinase (Shimada 1999), we introduce a shared reaction rate constant $k_{IKK\epsilon,TBK1}$ of kinase activation. We further assume the potential of pTBK1 and pIKK$\epsilon$ to induce IRF3 phosphorylation to be identical and introduce the shared reaction rate constant $k_{IRF3,IKK\epsilon,TBK1}$. These simplifications are necessary to prevent parameter non-identifiability arising from the semiquantitative western blot data. Once activated, IRF3 forms homodimers and is transported into the nucleus (Takahasi 2010). In our model, we neglect an explicit import reaction and assume phosphorylated IRF3 to be present only in the cell nucleus. Note, that we account for different volumes of the compartments in reactions that result in a compartmental change via the ratio of the compartmental volumes $V_{c2n}$ and $V_{n2c}$. $V_{c2n}$ corresponds to the ratio of the cytoplasmic volume to the nuclear volume and scales transport reactions into the nucleus, while $V_{n2c}$ corresponds to the ratio of nuclear to cytoplasmic volume and is included for transport reactions from the nucleus into the cytoplasm. The set of ODEs of the IRF3 branch of the RIGI pathway is given by:

$$\begin{matrix} \frac{d}{dt}\mathrm{IKK}\epsilon= & -k_{IKK\epsilon,TBK1}\cdot IKK\epsilon\cdot aMAVS+b_{\mathrm{deph}}\cdot pIKK\epsilon, \\ \frac{d}{dt}\mathrm{pIKK}\epsilon= & +k_{IKK\epsilon,TBK1}\cdot IKK\epsilon\cdot aMAVS-b_{\mathrm{deph}}\cdot pIKK\epsilon, \\ \frac{d}{dt}TBK1= & -k_{IKK\epsilon,TBK1}\cdot TBK1\cdot aMAVS+b_{\mathrm{deph}}\cdot pTBK1, \\ \frac{d}{dt}pTBK1= & +k_{IKK\epsilon,TBK1}\cdot TBK1\cdot aMAVS-b_{\mathrm{deph}}\cdot pTBK1, \\ \frac{d}{dt}IRF3= & -k_{IRF3,IKK\epsilon,TBK1}\cdot IRF3\cdot\left( \mathrm{pIKK}\epsilon+pTBK1 \right)+V_{n2c}\cdot b_{IRF3}\cdot{pIRF3}_{n}, \\ \frac{d}{dt}{pIRF3}_{n}= & +V_{c2n}\cdot k_{IRF3,IKK\epsilon,TBK1}\cdot IRF3\cdot\left( \mathrm{pIKK}\epsilon+pTBK1 \right)-b_{IRF3}\cdot{pIRF3}_{n}. \end{matrix}$$

In the NF-kappaB branch, IKKa and IKKb are activated at the mitochondrial signalling platforms and promote the formation of the IKK. To account for IKK activity, we use the essential phosphorylation of the main kinase IKKb as a marker for complex formation and incorporate a IKKb phosphorylation rate constant $k_{IKK\beta}$ and a dephosphorylation constant $b_{\mathrm{deph}}$ in our model. The activated IKKes (pIKK$\beta$) trigger the phosphorylation of IkB proteins, which are bound to cytoplasmic NF-kappaB (iNF$\kappa$B) and thereby promote the proteasomal degradation of IkB. The released NF-kappaB is subsequently phosphorylated and translocates into the cell nucleus (pNF-$\kappa$B${}_{n}$), where it induces the expression of IkB with the rate constant k${}_{\mathrm{IkB}\alpha}$. The proteasomal degradation of IkB, phosphorylation of NF-kappaB and its translocation are all described by a single reaction rate constant k${}_{\mathrm{NF}\kappa B}$. In the cell nucleus, phosphorylated NF-kappaB (pNF-$\kappa$B${}_{n}$) undergoes dephosphorylation with the rate constant $k_{deph,NF\kappa B}$ but still maintains its ability to produce IkB with the rate constant $k_{mRNA,IkB\alpha}$. Dephosphorylated nuclear NF-kappaB (NF-$\kappa$B${}_{n}$) is transported into the cytoplasm (NF-$\kappa$B${}_{c}$) with rate constant $k_{transp,NF\kappa B}$, where it is either bound by free IkB with the rate constant b${}_{I\kappa B\alpha}$ or reactivated by pIKK$\beta$ with the rate constant k${}_{\mathrm{NF}\kappa B}$. Additionally, degradation of IkB with the rate constant $\mu_{I\kappa B\alpha}$ is added. The NF-kappaB branch of the RIGI pathway is consequently given by:

$$\begin{matrix} \frac{d}{dt}\mathrm{IKK}\beta= & -k_{\mathrm{IKK}\beta}\cdot IKK\beta\cdot aMAVS+b_{\mathrm{deph}}\cdot pIKK\beta, \\ \frac{d}{dt}\mathrm{pIKK}\beta= & +k_{\mathrm{IKK}\beta}\cdot IKK\beta\cdot aMAVS-b_{\mathrm{deph}}\cdot pIKK\beta, \\ \frac{d}{dt}\mathrm{iNF}\kappa B= & -k_{\mathrm{NF}\kappa B}\cdot iNF\kappa B\cdot pIKK\beta+b_{I\kappa B\alpha}\cdot NF\kappa B_{c}\cdot I\kappa B\alpha, \\ \frac{d}{dt}\mathrm{pNF}\kappa B_{n}= & +V_{c2n}\cdot k_{\mathrm{NF}\kappa B}\cdot\left( \mathrm{iNF}\kappa B+NF\kappa B_{c} \right)\cdot pIKK\beta-k_{deph,NF\kappa B}\cdot pNF\kappa B_{n}, \\ \frac{d}{dt}\mathrm{NF}\kappa B_{n}= & +k_{deph,NF\kappa B}\cdot pNF\kappa B_{n}-k_{transp,NF\kappa B}\cdot NF\kappa B_{n}, \\ \frac{d}{dt}\mathrm{NF}\kappa B_{c}= & +V_{n2c}\cdot k_{transp,NF\kappa B}\cdot NF\kappa B_{n}-b_{I\kappa B\alpha}\cdot NF\kappa B_{c}\cdot I\kappa B\alpha-k_{\mathrm{NF}\kappa B}\cdot NF\kappa B_{c}\cdot pIKK\beta, \\ \frac{d}{dt}I\kappa B\alpha= & +k_{\mathrm{IkB}\alpha}\cdot\left( \mathrm{pNF}\kappa B_{n}+NF\kappa B_{n} \right)-b_{I\kappa B\alpha}\cdot NF\kappa B_{c}\cdot I\kappa B\alpha-\mu_{I\kappa B\alpha}\cdot I\kappa B\alpha. \end{matrix}$$

We further included the production of IFN$\beta$ mRNA under the transcriptional control by the IFN$\beta$ enhancosome, a complex that requires the interaction of activated IRF3, NF-kappaB and ATF2/c-jun heterodimers as a second order process in dependency of the nuclear IRF3 and NF-kappaB levels. IFN$\beta$ mRNA is further eliminated with the rate constant $\mu_{mRNA,IFN\beta}$, resulting in:

$$\frac{d}{dt}\mathrm{IFN}\beta_{\mathrm{mRNA}}=+V_{n2c}\cdot k_{mRNA,IFN\beta}\cdot pIRF3_{n}\cdot\left( \mathrm{pNF}\kappa B_{n}+NF\kappa B_{n} \right)-\mu_{mRNA,IFN\beta}\cdot IFN.\beta_{\mathrm{mRNA}}$$

|  |
| --- |

Figure 1.2: RIGI binds to cytoplasmic dsRNA and forms an active signalling complex (aRIGI) that induces the formation of mitochondrial signalling platforms (aMAVS). The kinases TBK1, IKKe and IKKb are recruited to the mitochondrial signalling platforms and are subsequently phosphorylated. While active TBK1 and IKKe (pTBK1 and pIKK$\epsilon$, respectively) induce the phosphorylation and subsequent translocation of IRF3 into the nucleus, triggers phosphorylated IKKb (pIKK$\beta$) the release and phosphorylation of the NF-kappaB subunit p65. The phosphorylated p65 translocates into the nucleus (pNF-$\kappa$B${}_{n}$) and induces IkB production. IkB is a feedback inhibitor and binds to free NF-kappaB in the cytoplasm (NF-$\kappa$B${}_{c}$) to form iNF-$\kappa$B${}_{c}$. Expression of IFN$\beta$ mRNA is further included under the regulation of phosphorylated IRF3 and nuclear NF-kappaB, two components of the IFN$\beta$ enhanceosome. Dotted lines indicate activated proteins, round boundaries indicate transcription factors and arrows correspond to reactions that are included in our mathematical model. Degradation rate constants are not included in this simplified model scheme.

### 1.2.2 Model parameters

RIG-I model parameters are divided into four categories: (i) Initial concentrations of model species, (ii) kinetic rate constants of biological processes, (iii) assay and cell properties and (iv) parameters to link simulated concentrations of a species to experimental data (scaling factors and background intensities). In the following, we introduce which assumptions and procedures are used to identify an optimal set of model parameters.

#### (i) Initial concentrations

Average protein copy numbers per cell are converted to concentrations (nM) by the reported cytoplasmic and nuclear volume of A549 cells (V${}_{\mathrm{cyt}}=1.2e-12$ L and V${}_{\mathrm{nucl}}=4.7e-13$ L (Jiang 2010)). We further assume an uniform distribution of dsRNA post electroporation in the complete assay, leading to intracellular initial dsRNA concentration of 1.95 nM. Our RIGI pathway model accounts for no pathway activation in the absence of dsRNA stimulation. Therefore, all initial levels of activated species are set to 0 nM and initial concentrations of inactivated proteins are equal to the total concentrations of proteins after accounting for their compartmental localization. A complete table of all initial species concentrations is provided in Table [1.1](#initConcCoreModel).

Table 1.1: Initial concentrations of the RIGI pathway model. Protein copy numbers were converted to [nM] by the cytoplasmic and nuclear volume of A549 cells (V${}_{cyt}=1.2e-12$ L and V${}_{nucl}=4.7e-13$ L, Jiang 2010) and under consideration of their compartmental localization. All concentrations of activated species are set to zero prior to stimulation.

| Species | [nM] | Compartment |  |
| --- | --- | --- | --- |
| RNA | 1.95 | cytoplasm |  |
| RIGI | 5.34 | cytoplasm |  |
| MAVS | 277.76 | cytoplasm |  |
| IKK$\epsilon$ | 3.08 | cytoplasm |  |
| TBK1 | 97.17 | cytoplasm |  |
| IRF3 | 37.86 | cytoplasm |  |
| IKK$\beta$ | 37.97 | cytoplasm |  |
| iNF-$\kappa$B | 11.36 | cytoplasm |  |
| I$\kappa$B$\alpha$ | 0 | nucleus |  |
| NF$\kappa$B${}_{c}$ | 101.73 | cytoplasm |  |
| IFN$\beta$ mRNA | 0 | cytoplasm |  |

#### (ii) Kinetic rate constants

As our model is quite detailed, including dsRNA sensing, RIGI pathway signalling and IFN$\beta$ mRNA production, we obtain structural and practical identifiability problems during the construction of our RIGI pathway model when optimizing all rate constants on our experimental data. Therefore, to reduce the degree of freedom, we fixed 9 kinetic rate constants based on reported values from literature ($\mu_{\mathrm{RNA}}$, $\mu_{\mathrm{RIGI}}$, $k_{syn,RIGI}$, $b_{I\kappa B\alpha}$, $\mu_{I\kappa B\alpha}$, $k_{\mathrm{RIGI}}$, $k_{\mathrm{MAVS}}$, $b_{\mathrm{MAVS}}$, $k_{mRNA,IFN\beta}$, see Table [1.2](#fixedRIGIRates)). The degradation rate constant $\mu_{\mathrm{RNA}}$ of double-stranded RNA in the cytoplasm of the cell is set to the fitted intracellular degradation rate constants of HCV dsRNA after the import in the cell by the same electroporation protocol (Binder, 2013). Reported half-lives for RIGI proteins in the human liver cancer cell line HepG2 are converted to the RIGI degradation rate constant ($\mu_{\mathrm{RIGI}}$) (Arimoto, 2007). The basal RIGI protein synthesis rate constant is consequently calculated using the cellular RIGI levels and the basal degradation rate constant, using:

$$k_{syn,RIGI}=\mathrm{RIGI}_{t=0}\cdot\mu_{\mathrm{RIGI}}.$$

The degradation rate constant of IkB and its binding rate constant to NF-kappaB are further taken from a mathematical model of NF-kappaB signalling by Hoffmann et al. (2002). Due to the lack of experimental data on the temporal RIGI activation and MAVS aggregation, we further use reported values for $k_{\mathrm{RIGI}}$, $k_{\mathrm{MAVS}}$ and $b_{\mathrm{MAVS}}$ from an existing mathematical model of the virus triggered type-I interferon signalling pathway (Zou, 2010). The elimination rate constant of IFN$\beta$ mRNA is further derived from its reported half-life in immortalized human bronchial epithelial cells (BEAS-2B cells):

$$\mu_{mRNA,IFN\beta}=\frac{\ln\left( 2 \right)}{4.16 h}\cdot\frac{1}{60 min/h}=0.0028 \min^{-1}.$$

The 11 remaining kinetic rate constants ($b_{IRF3}$, $b_{RIG-I}$, $b_{\mathrm{prot}}$, $k_{\mathrm{IKK}\beta}$, $k_{\mathrm{IKK}\epsilon,TBK1}$, $k_{IRF3}$, $k_{\mathrm{NF}\kappa B}$, $k_{deph,p65}$, $k_{I\kappa B\alpha}$, k${}_{transp,p65}$, k${}_{mRNA,IFN\beta}$) are optimized using the western blot and IFN$\beta$ data as described in Section [1.2.3](#SecParamOptCore). An overview over all fitted kinetic parameters and corresponding likelihood-based confidence intervalls is given in Table [1.3](#fittedRIGIRates).

Table 1.2: Fixed kinetic rate constants taken from literature. Rate constants are given in [1/min] or [1/(min$\cdot$nM)].

| Rate constant | Value | Reference |
| --- | --- | --- |
| $\mu_{\mathrm{dsRNA}}$ | 6.05e-3 | Binder et al. 2013 |
| $\mu_{RIG-I}$ | 0.0014 | Arimoto et al. 2007 |
| $\mu_{I\kappa B\alpha}$ | 0.0068 | Hoffmann et al. 2002 |
| $\mu_{mRNA,IFN\beta}$ | 0.0028 | Abe et al. 2012 |
| k${}_{syn,RIGI}$ | 0.0075 | this work |
| k${}_{RIG-I}$ | 0.0101 | Zou et al. 2010 |
| k${}_{\mathrm{MAVS}}$ | 0.009 | Zou et al. 2010 |
| b${}_{\mathrm{MAVS}}$ | 0.2189 | Zou et al. 2010 |
| $b_{I\kappa B\alpha}$ | 0.03 | Hoffmann et al. 2002 |

Table 1.3: Optimized reaction rate constants of the RIGI pathway model. Rate constants are given in [1/min] or [1/(min$\cdot$nM)]. The activation rate constant of IKKe and TBK1 (k${}_{IKK\epsilon,TBK1}$) was fixed after identifiability analysis (marked with *).

| Rate constant | Value | log10 Search Range | log10 95% CIs |
| --- | --- | --- | --- |
| b${}_{RIG-I}$ | 0.093 | [-4, +4] | [-1.47, -0.06] |
| b${}_{\mathrm{prot}}$ | 0.080 | [-4, +4] | [-1.52, -0.59] |
| k${}_{IRF3}$ | 0.017 | [-4, +2] | [-2.76, -1.30] |
| b${}_{IRF3}$ | 0.001 | [-4, +4] | [3.13, -2.84] |
| k${}_{\mathrm{IKK}\beta}$ | 0.048 | [-7, +3] | [-1.94, -0.77] |
| k${}_{\mathrm{NF}\kappa B}$ | 0.002 | [-5, +3] | [-2.89, -2.22] |
| k${}_{deph,NF\kappa B}$ | 0.052 | [-5, +3] | [-1.56, -0.98] |
| k${}_{I\kappa B\alpha}$ | 0.0001 | [-5, +3] | [-4.13, -3.88] |
| k${}_{transp,NF\kappa B}$ | 0.0002 | [-5, +3] | [-3.76, -3.38] |
| k${}_{mRNA,IFN\beta}$ | 1.65e${}^{-5}$ | [-7, +2] | [-5.01, -4.56] |
| k${}_{IKK\epsilon,TBK1}$ | 0.001* |  |  |

#### (iii) Assay and cell properties

To describe the RIGI activation in our *in vitro* experiment accurately, we derive important properties of the assay and A549 cells from the experimental protocol or literature. The cytoplasmic and nuclear volume of A549 cells (V${}_{\mathrm{cyt}}=1.2e-12$ L and V${}_{\mathrm{nucl}}=4.7e-13$ L) is derived from a morphometrical analysis by Jiang et al. (2010). We account for different sizes of compartments by considering the ratio between the ingoing- and outgoing volumes (V${}_{c2n}$, V${}_{n2c}$) to align with the law of mass conservation. The ratios are derived from the reported cytoplasmic and nuclear volumes (V${}_{c2n}=2.6$, V${}_{n2c}=0.39$). According to the experimental protocol, we further set the number of cells per well to $7e4$ and assume all cells in the assay to be equally affected by the electroporation.

Table 1.4: Properties of A549 cells and the experimental assay for dsRNA transfection experiments.

| Parameter | Value | Description |  |
| --- | --- | --- | --- |
| V${}_{\mathrm{cyt}}$ | 1.2e-12 [L] | Cytoplasmic volume |  |
| V${}_{\mathrm{nucl}}$ | 4.7e-13 [L] | Nuclear volume |  |
| V${}_{c2n}$ | 2.6 | Volume ratio: cytoplasm to nucleus |  |
| V${}_{n2c}$ | 0.39 | Volume ratio: Nucleus to cytoplasm |  |
| N${}_{\mathrm{cells}}$ | 7e4 [cells] | Number of cells in the assay |  |

#### (iv) Link between model species and experimental data

For the parameter optimization process, model species (in nM) are linked to experimentally observed normalized intensities (in au) by the use of scaling factors (SFs) and background parameters (bg). Background parameters account for background intensities in the experimental data. For species, which are not present prior to stimulation, background parameters are fixed to the mean of the normalized intensities for the first two time-points of the short time-period experiments (i.e,. 0  and 1 minutes post electroporation), assuming no noticeable changes during the first minute after electroporation. As observed IkB intensities at later time points are close to 0, we further neglect the background parameter for IkB and set bg${}_{I\kappa B\alpha}=0$. In addition to accounting for background noise in the experiments, scaling factors (nM/a.u.) are introduced to convert intracellular concentrations to measured intensities. The link between observables (highlighted with ${}_{m}$) and model species is consequently given by:

$$\begin{matrix} \mathrm{pIKK}\epsilon_{m}= & \frac{\mathrm{pIKK}\epsilon}{\mathrm{SF}_{\mathrm{pIKK}\epsilon}}+\mathrm{bg}_{\mathrm{pIKK}\epsilon}, \\ {pTBK1}_{m}= & \frac{pTBK1}{\mathrm{SF}_{pTBK1}}+\mathrm{bg}_{pTBK1}, \\ {pIRF3}_{m}= & \frac{pIRF3}{\mathrm{SF}_{pIRF3}}+\mathrm{bg}_{pIRF3}, \\ \mathrm{pIKK}\beta_{m}= & \frac{\mathrm{pIKK}\beta}{\mathrm{SF}_{\mathrm{IKK}\beta}}+\mathrm{bg}_{\mathrm{IKK}\beta}, \\ \mathrm{pNF}\kappa B_{m}= & \frac{\mathrm{pNF}\kappa B}{\mathrm{SF}_{\mathrm{NF}\kappa B}}+\mathrm{bg}_{\mathrm{mNF}\kappa B}, \\ I\kappa B_{m}= & \frac{\mathrm{mI}\kappa B+iNF\kappa B}{\mathrm{SF}_{\mathrm{mI}\kappa B}}+\mathrm{bg}_{\mathrm{mI}\kappa B}, \\ \mathrm{IFN}\beta_{mRNA,m}= & \mathrm{IFN}\beta_{\mathrm{mRNA}}+\mathrm{bg}_{\mathrm{IFN}\beta,mRNA}. \end{matrix}$$

Scaling factors as well as the background parameter for IFN$\beta$ are optimized during the parameter optimization process (see Section [1.2.3](#SecParamOptCore)). Resulting scaling factors and background parameters of the RIG-I pathway model are given in Table [1.5](#ScaleRIGICore).

Table 1.5: Scaling factors and background parameters to link intracellular concentrations (nM) to observed intensities (a.u.) in the RIG-I pathway model. Parameters that are obtained directly from experimental data as described in the main text are highlighted (*).

| Observable | scaling factor | background parameter |  |
| --- | --- | --- | --- |
| pIKK$\epsilon_{m}$ | 0.1 | 0.557* |  |
| pTBK1${}_{m}$ | 4.09 | 0.610* |  |
| pIRF3${}_{m}$ | 80.25 | 0.059* |  |
| pIKK$\beta_{m}$ | 0.38 | 0.404* |  |
| pNF-$\kappa$B${}_{m}$ | 62.31 | 0.924* |  |
| I$\kappa$B$\alpha_{m}$ | 51.64 | 0* |  |
| $\mathrm{mIFN}\beta_{mRNA,m}$ | - | 0.012 |  |

### 1.2.3 Parameter optimization

We calibrate the mathematical model by minimizing the negative logarithm of the likelihood function for the observed experimental data given our model parameters. We employ a global optimization method using a multi-start approach based on LHS, which is implemented in the Data2Dynamics framework for MATLAB (Raue 2013, Raue 2015). All open parameters are optimized on log-scale as recommended in case of non-negative parameters with unknown order of magnitude (Raue 2013). The search space of all open parameters further spanned multiple orders of magnitude (see Table [1.2](#fixedRIGIRates)). To ensure convergence towards the global minimum of the multi-dimensional search space, 1500 deterministic optimisation runs (LSQNONLIN) are peformed. The final goodness of fit, i.e. the difference between the likelihood of a run compared to the best obtained likelihood among all runs, is shown in Figure [1.3](#FigCoreModelConv) for every optimization run. It demonstrates several local optima and repeated convergence towards the global optimum, indicating reliable detection of the global optimum.

|  |
| --- |

Figure 1.3: The difference between the best value of the likelihood function for every run and the best likelihood value among all runs (goodness of fit).

### 1.2.4 Model fit

The model fit of the RIGI pathway model is given in Figure [1.4](#ModelFitCoreModel) for the first 240 minutes post electroporation alongside the experimental data. Note that, short time-period (up to 4 h.p.e.) and long time-period (up to 24 h.p.e.) experiments are considered in the fitting process. Incorporating the long time-period experiments is necessary to accurately capture the late induction of IkB, which was underestimated when only considering the short time-period experiments.

|  |
| --- |

Figure 1.4: Experimental data (points) and model simulations of the RIGI pathway model for the optimized parameter set. The dynamics of the IRF3 branch and the NF-kappaB branch as well as IFN$\beta$ mRNA levels are accurately captured by our model.

### 1.2.5 Identifiability analysis

To detect structural and practical identifiability problems, we derive the profile likelihood estimates as described previously and implemented in the Data2Dynamics framework (Raue 2009, Raue 2015). In short, the method detects functionally related model parameters and identifies insufficient experimental evidence for the given model complexity. Starting from the optimal set of parameters (the global optimum), every parameter $i$ is stepwise fixed at increasingly differing values, while all other open parameters are refitted. The decrease in fitting quality over changes in the parameter $i$ allows to identify likelihood-based 95% confidence intervals. As we observed a linear dependency between $k_{IKK\epsilon,TBK1}$ and $k_{IRF3,IKK\epsilon,TBK1}$, when optimizing all open parameters on the experimental data, $k_{IKK\epsilon,TBK1}$ was fixed. This resulted in the likelihood profiles in Figure [1.5](#RIGIcorePLE) for our RIGI signalling model. The analysis reveals no further structural or practical non-identifiability of the optimized model parameters. The derived confidence intervals together with the optimal value and search range of every parameter is given in Table [1.3](#fittedRIGIRates).

|  |
| --- |

Figure 1.5: Profile likelihood estimates of optimized parameters in the RIGI signalling model. Blue points indicate the parameter value of the best fit, blue error bars visualize the 95% confidence intervals defined as the intercept of the likelihood profile and the statistical threshold indicated by a red line. Parameter values and confidence intervals are listed in Table [1.3](#fittedRIGIRates).

## 1.3 Model extension: Antiviral innate immune response model

In the following, we introduce the published mathematical model for JAK-STAT signalling by Maiwald et al. (2010) and our strategy to couple it to our developed RIGI pathway model.

### 1.3.1 Model coupling and extension

To create a detailed, mechanistic model of the antiviral innate immune response, we aim to combine our RIGI pathway model with the existing JAK-STAT mode by Maiwald et al. (2010). The coupled model should capture the initiation of RIGI signalling and IFN expression, but also the reinforcement of the antiviral state via the JAK-STAT pathway. In the model coupling process, the dynamics of IFN is crucial: IFN is produced by the RIGI pathway and subsequently triggers JAK-STAT signalling. Consequently, we can derive a link between the two models by assessing the IFN dynamics upon stimulation with dsRNAs. The RIGI pathway model includes the production of IFN$\beta$ mRNA, a type-I IFN just as IFN$\alpha$, by phosphorylated IRF3 and nuclear NF-kappaB. Therefore, we define the produced type-I mRNA as template for the production of IFN$\alpha$ in our model. As in the original JAK-STAT model, we divide the total IFN in the assay into two IFN species, here termed IFN${}_{\mathrm{pre}}$ (IFN added to stimulate the cells) and IFN${}_{\mathrm{free}}$ (IFN which is in close proximity to interferon receptors and can activate JAK-STAT signalling). The new set of ODEs describing the rate of change of IFN$\alpha$ is given by

$$\begin{matrix} \frac{d}{dt}\mathrm{IFN}_{\mathrm{pre}}= & +k_{\beta,IFN}\cdot\mathrm{IFN}_{\mathrm{mRNA}}-k_{68}\cdot\mathrm{IFN}_{\mathrm{pre}}-\mu_{\mathrm{IFN}}\cdot\mathrm{IFN}_{\mathrm{pre}}, \\ \frac{d}{dt}\mathrm{IFN}_{\mathrm{free}}= & +k_{68}\cdot\mathrm{IFN}_{\mathrm{pre}}+k_{6}\cdot\mathrm{IFNAR}_{\mathrm{dimer}}-k_{5}\cdot\mathrm{IFN}_{\mathrm{free}}\cdot Receptor:JAK\cdot Receptor:TYK. \end{matrix}$$

##### IFN$\alpha$ as link between both pathways

We included the production of IFN${}_{\mathrm{pre}}$ by IFN$\beta$ mRNA and account for a potential degradation of IFN${}_{\mathrm{pre}}$. The remaining terms in the ODEs are identical to the ones published by Maiwald et al. (2010). In short, $k_{68}$ corresponds to the transition from IFN${}_{\mathrm{pre}}$ to IFN${}_{\mathrm{free}}$, $k_{6}$ to dissociation of IFN from the IFNAR receptor and $k_{5}$ to IFNAR activation. Protein complexes are highlighted by linking single complex components via ":". For a complete overview of reactions in the published JAK-STAT model, we refer to the original work.

##### Experimental setup-specific differences

It is important to point out that the cells used to investigate IFN signaling in the Maiwald study (Huh7.5) are different from the A549 cells used to study the RIGI pathway. To account for deviations due to differences in the cell types, we readjust total protein levels in the JAK-STAT model according to proteomics data of Huh7.5 cells (Table [1.7](#TabJAKSTATcompInitialVal)). We further account for differences in the experimental setup, by readjusting the rate constant k${}_{68}$ describing the transition of IFN to a close proximity of the IFN receptors on our experimental data (see Section [1.3.2](#SecCouplModelParam)). All other species from the original JAK-STAT model are represented by the same ODEs and all kinetic parameters are taken from the original work.

##### ISG expression

To account for cross talk or feedback between the pathways as well as for markers of an antiviral state in the cell, we further incorporate the expression of different classes of ISGs into the model. In total, we consider the expression of 6 genes in our model (CCL5, CXCL10, IFIT1, IFN$\lambda$, RIGI, Mx1), in addition to the already existing upregulated species IkB, IFN$\beta$, IRF9 and SOCS. The genes are selected to either study their role as a feedback regulators, or as markers of the antiviral gene expression.

Table 1.6: Newly included ISGs and their respective regulator in our coupled innate immune response model.

| Protein | Transcription factors | Reference |  |
| --- | --- | --- | --- |
| CCL5 | IRF3 | Liu 2005 |  |
| CXCL10 | IRF3 | - |  |
| IFIT1 | IRF3, ISGF3 | Fensterl 2011 |  |
| IFN$\lambda$ | IRF3 | Hemann 2017, Lazear 2015 |  |
| RIG-I | IRF3, ISGF3 | Kim 2008 |  |
| MX1 | IRF3, ISGF3 | Verhelst 2013 |  |

Depending on the availability of qPCR and western blot data, ISGs are included at the transcript level (CCL5, CXCL10), the protein level (RIGI) or at both levels (IFIT1, IFN$\lambda$, Mx1) in our model. The expression of mRNA is thereby dependent on the active concentration of the proposed transcription factor and implemented as mass-action kinetic reaction. Furthermore, we account for a first order decay of mRNAs (denoted $\mu$) and, when a protein species is included into the model, a translation and protein degradation process. ISG expression on mRNA level is consequently described by the following set of ODEs:

$$\begin{matrix} \frac{d}{dt}\mathrm{CCL}5_{\mathrm{mRNA}}= & V_{n2c}\cdot k_{CCL5,mRNA}\cdot{pIRF3}_{n}-\mu_{CCL5,mRNA}\cdot{CCL5}_{mRNA}, \\ \frac{d}{dt}\mathrm{CXCL}{10}_{\mathrm{mRNA}}= & V_{n2c}\cdot k_{CXCL10,mRNA}\cdot{pIRF3}_{n}-\mu_{CXCL10,mRNA}\cdot{CXCL10}_{mRNA}, \\ \frac{d}{dt}\mathrm{IFIT}1_{\mathrm{mRNA}}= & V_{n2c}\cdot k_{IFIT1,mRNA}\cdot OCBS+V_{n2c}\cdot k_{IRF3,mRNA}\cdot{pIRF3}_{n}-\mu_{IFIT,mRNA}\cdot IFIT1_{\mathrm{mRNA}}, \\ \frac{d}{dt}\mathrm{IFN}\lambda_{\mathrm{mRNA}}= & V_{n2c}\cdot k_{IFN\lambda,mRNA}\cdot{pIRF3}_{n}-\mu_{IFN\lambda,mRNA}\cdot IFN\lambda_{\mathrm{mRNA}}, \\ \frac{d}{dt}\mathrm{MX}1_{\mathrm{mRNA}}= & V_{n2c}\cdot k_{MX1,mRNA}\cdot OCBS+V_{n2c}\cdot k_{MX1,pIRF3}\cdot pIRF3-\mu_{MX1}\cdot MX1_{\mathrm{mRNA}}. \end{matrix}$$

The species OCBS corresponds to occupied (i.e. active) ISGF3 binding sites, which drive JAK-STAT dependent gene expression. As mentioned beforehand, we further included the PRR RIGI and the two well studied antiviral effectors IFIT1 and Mx1 at the protein level. While IFIT1 and Mx1 was included to assess the post-transcriptional regulation of antiviral effectors, RIGI protein upregulation can play an important role in case of reinfection events by increasing the sensitivity of dsRNA detection. The modified ODE for RIGI and the newly established ones for IFIT1 and Mx1 are given by:

$$\begin{matrix} \frac{d}{dt}RIG-I= & -k_{\mathrm{RIGI}}\cdot dsRNA\cdot RIGI+b_{\mathrm{RIGI}}\cdot aRIGI-\mu_{\mathrm{RIGI}}\cdot RIGI \\ & +k_{syn,RIGI}+k_{transl,ISGs}\cdot MX1_{\mathrm{mRNA}}, \\ \frac{d}{dt}IFIT1= & k_{transl,IFIT1}\cdot IFIT1_{\mathrm{mRNA}}-\mu_{IFIT}\cdot IFIT1, \\ \frac{d}{dt}MX1= & k_{transl,ISGs}\cdot MX1_{\mathrm{mRNA}}-\mu_{MX1}\cdot MX1. \end{matrix}$$

Note we have no RIGI mRNA in the model as no experimental qPCR data are available. For simplicity, we model RIGI protein production as dependent on the Mx1 mRNA levels. Mx1 is a gene that is, likewise to RIGI, regulated via both signalling pathways of the antiviral innate immunity. This assumption is sufficient to describe the observed RIGI protein dynamics (see Figure [1.8](#ModelFitCoupledModel)). Of interest, the expression of RIGI and Mx1 is explained by a single shared translation rate constant $k_{transl,ISGs}$.
To assess the amount of functional type-III interferon, we further incorporated IFN$\lambda$ on the protein level using

$$\frac{d}{dt}IFN\lambda_{mRNA}=k_{trans1,IFN\lambda}\cdot IFN\lambda_{mRNA}-\mu_{IFN\lambda}\cdot IFN\lambda_{mRNA}.$$

In total eight new species are introduced in this section (CCL5${}_{\mathrm{mRNA}}$, CXCL10${}_{\mathrm{mRNA}}$, IFIT1${}_{\mathrm{mRNA}}$, IFIT1, IFN$\lambda_{\mathrm{mRNA}}$, IFN$\lambda$, MX1${}_{\mathrm{mRNA}}$, MX1) and the ODEs of two existing species are modified with respect to the RIGI pathway or JAK-STAT model by Maiwald et al. 2010 (IFN${}_{\mathrm{pre}}$, RIGI) . Our detailed mathematical model of the cell-intrinsic antiviral innate immune response comprises 66 unique species. A simplified representation of the complete model is depicted in Figure [1.6](#coupledModelOverview) alongside with an overview about the incorporated gene expression.

*Figure 1.6: Simplified representation of the developed cell-intrinsic innate immune response model. The innate immune response is triggered upon sensing of dsRNA, initiates RIG-I pathway signalling and interferon expression (left part). The produced interferon activates a model of JAK-STAT signalling which structure and parameters were published in Maiwald 2010. We further extended the model to include ISG expression under the regulation of transcription factors within our model (feedback regulators, antiviral effectors).*

### 1.3.2 Model parameters

Combining and extending the RIGI pathway and the JAK-STAT model requires the estimation of additional parameters. Likewise to the previous section, we distinguish between: (i) Initial concentrations, (ii) kinetic rate constants, (iii) assay and cell properties and (iv) parameters to link simulated concentrations of a species to experimental observations.

##### (i) Initial concentrations

Since ISGs are absent or present in small amounts without stimulation, we assume negligible amounts at the start of the simulation and define them as zero prior to dsRNA electroporation (Table [1.8](#initConcISGs)). Unlike to the original work by Maiwald et al. (2010), no IFN stimulation is used in our set-up, and the initial IFN concentration is set to zero. Initial values for further components of the JAK-STAT signalling cascade were adjusted based on the reported absolute protein copy numbers in A549 cells and converted to [nM] by using the reported cytoplasmic and nuclear volumes of A549 cells. For species without reported copy numbers in the proteomics data (i.e. IFNAR1, IFNAR2, IRF9) or where no specific protein is referred to in the JAK-STAT model (i.e. cytoplasmic phosphatase, nuclear phosphatase, number of transcription factor binding sites on the DNA), we adopted the initial values from Maiwald et al. (2010). Basal levels of JAK-STAT model species are determined by simulating their steady-state concentrations in absence of any stimulus.

Table 1.7: Cellular protein concentrations of JAK-STAT pathway components prior to stimulation with dsRNA.

| Species | Total levels [nM] | Reference |
| --- | --- | --- |
| IFNAR1 | 1000 | Maiwald 2010 |
| IFNAR2 | 1000 | Maiwald 2010 |
| JAK | 151.87 | A549 proteomics data |
| TYK | 20.70 | A549 proteomics data |
| STAT1 | $1.12e3$ | A549 proteomics data |
| STAT2 | 6.50 | A549 proteomics data |
| IRF9 | 45 | Maiwald 2010 |
| CP | 20 | Maiwald 2010 |
| NP | 40 | Maiwald 2010 |
| PIAS | 41.96 | A549 proteomics data |
| TFBS | 500 | Maiwald 2010 |

Table 1.8: Initial concentrations of the newly introduced species. All newly introduced species and IFN$\alpha$ are set to zero prior to stimulation.

| Species | [nM] | Compartment |
| --- | --- | --- |
| IFN${}_{\mathrm{pre}}$ | 0 | cytoplasm |
| CCL5${}_{\mathrm{mRNA}}$ | 0 | cytoplasm |
| CXCL10${}_{\mathrm{mRNA}}$ | 0 | cytoplasm |
| IFIT1${}_{\mathrm{mRNA}}$ | 0 | cytoplasm |
| IFIT1 | 0 | cytoplasm |
| IFN$\lambda_{\mathrm{mRNA}}$ | 0 | cytoplasm |
| IFN$\lambda$ | 0 | cytoplasm |
| MX1${}_{\mathrm{mRNA}}$ | 0 | cytoplasm |
| MX1 | 0 | cytoplasm |

##### (ii) Kinetic rate constants

In total 22 new rate constants are introduced, and one additional rate constant from the Maiwald model is readjusted to account for differences in the transition from IFN${}_{\mathrm{pre}}$ to IFN${}_{\mathrm{free}}$ ($k_{68}$). Among those 22 parameters, 8 degradation rate constants are fixed based on reported half-lives in literature for CCL5${}_{\mathrm{mRNA}}$, CXCL10${}_{\mathrm{mRNA}}$, IFIT1${}_{\mathrm{mRNA}}$, IFIT1, IFN$\lambda_{\mathrm{mRNA}}$, IFN$\lambda$, MX1${}_{\mathrm{mRNA}}$ and MX1.

The effect of a degradation of IFN$\alpha$ in the supernatant of *in vitro* assays could not be identified and no loss in the fitting quality was obtained when neglecting IFN${}_{\mathrm{pre}}$ degradation. Therefore, we further set $\mu_{\mathrm{IFN}}=0 h^{-1}$. A list of all newly introduced parameters that are fixed according to literature information is given in Table [1.10](#fittedcoupledRIGIRates).
The remaining 13 rate constants are adjusted during the fitting process on the experimental data as described in Section [1.3.3](#ParamOptCoupl). A list of all optimized parameters, the search range and confidence intervals is given in Table [1.10](#fittedcoupledRIGIRates).

Table 1.9: Fixed degradation rate constants according to literature. Rate constants are given in [1/min] and were derived using the reported half-life (HL).

| Rate constant | Value | Reference | Note |
| --- | --- | --- | --- |
| $\mu_{CCL5,mRNA}$ | 0.0012 | Marccais et al. 2006 | HL~ 10 h |
| $\mu_{CXCL10,mRNA}$ | 0.0067 | Dhillon et al. 2007 | HL~ 43-103 min |
| $\mu_{IFIT1,mRNA}$ | 0.0052 | Sharova et al. 2009 | mean HL~ 2.23 h |
| $\mu_{IFIT1}$ | 4.8$e^{-4}$ | Schmid et al. (2015) | HL > 24 h |
| $\mu_{IFN\lambda,mRNA}$ | 0.0036 | Schmidtke et al. 2018 | HL~ 3.25 h |
| $\mu_{IFN\lambda}$ | 1.6$e^{-4}$ | Voigt et al. 2015 | HL > 72 h |
| $\mu_{MX1,mRNA}$ | 0.0028 | Lam 2001 | HL ~ 4.14 h |
| $\mu_{MX1}$ | 2.1$e^{-4}$ | Schmid et al. (2015) , Ronni et al. (1993) | HL ~ 2.3 days |
| $\mu_{\mathrm{IFN}}$ | 0 | - | HL ~ 97h |

Table 1.10: Optimized reaction rate constants during the model extension process. Rate constants are given in [1/min] or [1/(min$\cdot$nM)], respectively.

| Rate constant | Value | log10 Search range | log10 95% CIs |
| --- | --- | --- | --- |
| k${}_{68}$ | $4.1e-4$ | [-5,3] | [-Inf, -2.83] |
| k${}_{\mathrm{IFN}}$ | $6.0e-5$ | [-5,3] | [-Inf, -2.75] |
| k${}_{IRF3,IFIT1}$ | $0.35$ | [-5,3] | [-1.94, 0.64] |
| k${}_{IFIT1,mRNA}$ | $63.62$ | [-8, 3] | [-0.26, Inf] |
| k${}_{CCL5,mRNA}$ | $9.5e-4$ | [-5,3] | [-4.9, -1.6] |
| k${}_{CXCL10,mRNA}$ | $0.022$ | [-5,3] | [-3.29,-0.44] |
| k${}_{IFN\beta,mRNA}$ | $2.0e-4$ | [-5,3] | [-Inf, -2.74] |
| k${}_{IFN\lambda,mRNA}$ | $0.15$ | [-5,3] | [-Inf, 0.38] |
| k${}_{MX1,mRNA}$ | $15.41$ | [-5,3] | [-0.74, Inf] |
| k${}_{MX1,pIRF3}$ | $0.0070$ | [-5,3] | [-3.7, -1.0] |
| k${}_{transl,ISGs}$ | $1.2e-4$ | [-5,3] | [-Inf -2.14] |
| k${}_{transl,IFIT1}$ | $3.4e-5$ | [-5,3] | [-Inf, -1.26] |
| k${}_{transl,IFN\lambda}$ | $6.2e-4$ | [-5,3] | [-4.40, Inf] |

#####

##### (iii) Additional parameters

In the work by Maiwald and colleagues, IFN$\alpha$ is administered in U/mL and converted to nM. We apply the same scaling factor (here named SF${}_{nM2IU}$) to describe the link between IFN concentrations in nM and the corresponding experimentally determined IFN levels. A IFN concentration of 500 IU/mL corresponds to 6.5 nM IFN in the original work, yielding

$$\mathrm{SF}_{nM2IU}=\frac{500}{6.5}=76.92,$$

which is multiplied with IFN concentrations in the model to obtain U/mL values. Experimentally observed levels of IFN$\lambda$ are further quantified in pg/mL. We therefore convert nM to pg/mL by introducing the scaling factor:

$$\mathrm{SF}_{nM2pgmL}=\frac{{10}^{-9}\frac{\mathrm{mol}}{L}\cdot6.022\cdot{10}^{23}\frac{\mathrm{molecules}}{\mathrm{mol}}\cdot3.7\cdot{10}^{-11}\frac{\mathrm{pg}}{\mathrm{molecule}}}{1e3\frac{\mathrm{mL}}{L}}=22.28\frac{pg/mL}{\mathrm{nM}}.$$

Table 1.11: Derived scaling factors to link model species to experimentally observed IFN concentrations.

| Parameter | Value | Description |
| --- | --- | --- |
| SF${}_{nM2IU}$ | 76.92 [IU/mL / nM] | Converts IFN$\alpha$ from nM to IU/mL |
| SF${}_{nM2pgmL}$ | 22.28 [pg/mL / nM] | Converts IFN$\lambda$ from nM to pg/mL |

#####

##### (iv) Linking model species to experimental data

For the parameter optimization problem, we first have to generate a link between the new experimental data (e.g. western blot, qPCR data) and our model species. Similar to our approach for the RIGI pathway model, we introduce scaling factors (SF) and background parameters (bg). The corresponding link for IFN and western blot data is given by

$$\begin{matrix} \mathrm{IFN}\alpha_{m}= & \mathrm{IF}N_{\mathrm{pre}}\cdot\mathrm{SF}_{nM2IU}, \\ \mathrm{IFN}\lambda_{m}= & \mathrm{IFN}\lambda\cdot\mathrm{SF}_{nM2pg}, \\ {IFIT1}_{m}= & \frac{IFIT1}{\mathrm{SF}_{IFIT1}}+bg_{IFIT1}, \\ {IRF9}_{m}= & \frac{{IRF9}_{\mathrm{tot}}}{\mathrm{SF}_{IRF9}}+\mathrm{bg}_{IRF9}, \\ {MX1}_{m}= & \frac{MX1}{\mathrm{SF}_{MX1}}+bg_{MX1}, \\ {pSTAT2}_{m}= & \frac{{pSTAT2}_{\mathrm{tot}}}{\mathrm{SF}_{pSTAT2}}+\mathrm{bg}_{pSTAT2} \\ \mathrm{RIGI}_{m}= & \frac{RIGI+aRIGI}{\mathrm{RIGI}_{0}}, \end{matrix}$$

whereby observables are highlighted by ${}_{m}$. Note, relative data of total RIGI levels are available, resulting in a different scaling. The background parameters for ${IFIT1}_{m}$, ${IRF9}_{m}$ and ${MX1}_{m}$ are fixed based on the experimental data, whereas the background parameter for phosphorylated STAT2 (${pSTAT2}_{m}$) is adjusted during the model optimization process alongside all SFs.
Whenever multiple model species contribute to the observable, e.g. multiple forms of phosphorylated STAT2 are present in the model but we are limited to information about total phosphorylated STAT2 in the cell, concentrations of all contributing species are considered. Therefore, we weight the concentrations of a species by the volume of the corresponding compartment and subsequently divide the derived amount by the cellular volume. As the total phosphorylated STAT2 levels and the total IRF9 protein level are dependent on multiple model species of the JAK-STAT signalling cascade, we define the total phosphorylated STAT2 levels per cell (pSTAT2${}_{\mathrm{tot}}$) as:

$$\begin{matrix} {pSTAT2}_{\mathrm{tot}}= & \frac{V_{\mathrm{cyt}}\cdot\left( ISGF_{3c}+STAT1c*:STAT2c*+ISGF_{3c}:CP+STAT1c*:STAT2c*:CP \right)}{V_{cell}} \\ + & \frac{V_{\mathrm{nucl}}\cdot\left( STAT1n*:STAT2n*+ISGF_{3n}+STAT1n*:STAT2n*:NP+ocBS:NP \right)}{V_{cell}} \\ + & \frac{V_{\mathrm{nucl}}\cdot\left( ocBS+PIAS:ISGF_{3n}+ISGF_{3n}:NP \right)}{V_{cell}} \end{matrix}$$

and the total IRF9 protein levels within a cell (${IRF9}_{\mathrm{tot}}$) as:

$$\begin{matrix} {IRF9}_{\mathrm{tot}}= & \frac{V_{\mathrm{cyt}}\cdot\left( IRF9c+STAT2c:IRF9+ISGF_{3c}+ISGF_{3c}:CP \right)}{V_{cell}} \\ + & \frac{V_{\mathrm{nucl}}\cdot\left( IRF9n+ISGF_{3n}+ocBS:NP+ocBS+PIAS:ISGF_{3n}+STAT2n:IRF9 \right)}{V_{cell}} \\ + & \frac{V_{\mathrm{nucl}}\cdot\left( ISGF_{3n}:NP \right)}{V_{cell}}. \end{matrix}$$

Activated species are marked ("$*$") and protein complexes are represented by the name of their components linked with ":".
Transcript levels are also transformed to intracellular concentrations. We introduce a shared scaling factor SF${}_{\mathrm{mRNA}}$ and a species-specific parameter accounting for background intensities (bg). We chose a shared scaling factor as absolute quantification qPCR experiments are performed resulting in mRNA counts that are comparable between transcripts. The link between mRNA observables and model species is consequently

$$\begin{matrix} \mathrm{CCL}5_{mRNA,m}= & \mathrm{CCL}5_{\mathrm{mRNA}}\cdot SF_{\mathrm{mRNA}}+bg_{CCL5,mRNA}, \\ \mathrm{CXCL}{10}_{mRNA,m}= & \mathrm{CXCL}{10}_{\mathrm{mRNA}}\cdot SF_{\mathrm{mRNA}}+bg_{CXCL10,mRNA}, \\ {IFIT1}_{mRNA,m}= & {IFIT1}_{\mathrm{mRNA}}\cdot\mathrm{SF}_{\mathrm{mRNA}}+bg_{IFIT1,mRNA}, \\ \mathrm{IFN}\lambda_{mRNA,m}= & {IFN\lambda}_{\mathrm{mRNA}}\cdot\mathrm{SF}_{\mathrm{mRNA}}+bg_{IFN\lambda,mRNA}, \\ {MX1}_{mRNA,m}= & {MX1}_{mRNA\cdot}\mathrm{SF}_{\mathrm{mRNA}}+bg_{MX1,mRNA}. \end{matrix}$$

The mRNA scaling factor SF${}_{\mathrm{mRNA}}$ and all background parameters for mRNA species are adjusted during the parameter optimization process. An overview of all SFs and background parameters is given in Table [1.12](#ScaleRIGICoupled).

Table 1.12: Scaling factors and background parameters to link species of the coupled innate immune response model to experimental observables.

| Observable | SF | Background | Note |
| --- | --- | --- | --- |
| IFIT1${}_{m}$ | 21 | 1* | *fixed |
| IFN-$\alpha_{m}$ | 76.9 | - |  |
| IFN-$\lambda_{m}$ | 22.28 | - |  |
| IRF9${}_{m}$ | 16.9 | 0* | *fixed |
| MX1${}_{m}$ | 9.87 | 0* | *fixed |
| RIG-I${}_{m}$ | - | - | relative to initial RIGI levels |
| pSTAT2${}_{m}$ | 0.045 | 0.016 |  |
| CCL5${}_{mRNA,m}$ | 0.21 | 0.01 |  |
| CXCL10${}_{mRNA,m}$ | 0.21 | 0.0007 |  |
| IFIT1${}_{mRNA,m}$ | 0.21 | 0.52 |  |
| IFN$\lambda_{mRNA,m}$ | 0.21 | 0.0072 |  |
| MX1${}_{mRNA,m}$ | 0.21 | 0.091 |  |

###

### 1.3.3 Parameter optimization

The negative logarithm of the likelihood function is minimized for the experimental data and our model topology to find the best set of parameters. Importantly, during the fitting process, parameters of the RIGI pathway model are not optimized but kept constant as listed in Table [1.3](#fittedRIGIRates) and Table [1.2](#fixedRIGIRates). Once again, a global optimization algorithm based on LHS as implemented in the Data2Dynamics toolbox is applied. Prior to optimization, experimentally measured mRNA levels are log10-transformed. Likewise, all open parameters are log10-transformed prior to optimization within a search space of at least six orders of magnitude. We performed 1000 independent deterministic optimization runs to ensure convergence towards the global optimum. Figure [1.7](#PlotFitsCoupledModel) shows the local and global optima for all performed optimization runs and demonstrates repeated convergence towards a single optimum.

|  |
| --- |

Figure 1.7 Goodness of fit, i.e. the difference between the likelihood of a run compared to the best obtained likelihood among all runs, for every deterministic optimization run for the coupled innate immune response model. We performed 1000 independent optimization runs starting from deviating initial parameter estimates.

### 1.3.4 Model fit

The experimental data and our best model fit are shown in Figure [1.8](#ModelFitCoupledModel).

|  |
| --- |

Figure 1.8: Model fit of the coupled cell-intrinsic innate immune response model. A549 wt (black) or IFN$\alpha/\lambda$ double receptor knock-out A549 cells (red) are stimulated by dsRNA. Resulting experimental data (circles) are shown together with the model simulations (lines) for the optimized set of model parameters. Sensing of dsRNA results in the activation of IRF3 and NF-kappaB signalling (left side), which triggers IFN expression, JAK-STAT signalling and ISG expression (right side).

### 1.3.5 Identifiability analysis

Once again a structural and practical identifiability analysis is performed to detect related model parameters and check for insufficient experimental data. The resulting profile likelihood for every optimized parameter is shown in Figure [1.10](#FigPLECoupledModel). While most of the model parameters are identifiable and likelihood-based confidence intervals are obtained (15 out of 25), we identify only a single boundary for 10 model parameters. For an overview of the resulting profile likelihoods, we refer to Figure [1.10](#FigPLECoupledModel). Likelihood based confidence intervals are given together with the optimal set of model parameters in Table [1.10](#fittedcoupledRIGIRates).

## 1.4 Mode of action of viral antagonists on immune signalling

CSFV Npro is reported to induce proteasomal degradation of IRF3 (Bauhofer 2007). As our cells stably express CSFV Npro prior to stimulation and IRF3 levels decrease in CSFV Npro-dependent manner, less IRF3 is present at the time-point of electroporation compared to A549 *wt* cells. Consequently, the effect of CSFV Npro is incorporated into our model by reducing the concentration of IRF3 using

$$IRF3={IRF3}_{0}\cdot\frac{N_{\mathrm{pro}}}{K_{M,Npro}+N_{\mathrm{pro}}},$$

whereas $K_{M,Npro}$ corresponds to the CSFV Npro concentration of half-maximal IRF3 down regulation and IRF3${}_{0}$ to the IRF3 level in *wt* A549 cells.

DENV NS5 inhibits the cell-intrinsic immunity by provoking the proteasomal degradation of the cellular STAT2 protein pool (Morrison 2013). Since DENV NS5 is present prior to stimulation, we again assume the STAT2 levels in DENV NS5-expressing cells to be in steady state at the time-point of electroporation and model the relation between the STAT2 and DENV NS5 using

$$STAT2={STAT2}_{0}\cdot\frac{K_{M,NS5}}{K_{M,NS5}+NS5}.$$

HCV encodes the HCV NS3/4A, which cleaves MAVS off the mitochondrial membrane and thereby renders a key signalling protein in the RIGI pathway non-functional (Li 2005, Lin 2006, Meylan 2005). HCV NS3/4A expression consequently results in decreased functional MAVS levels:

$$MAVS=\mathrm{MAVS}_{0}\cdot\frac{K_{M,NS34A}}{K_{M,NS34A}+NS34A}.$$

Unlike to the other tested viral antagonists, HCV NS3/4A is stably expressed in A549 cells under the regulation of a single promoter. To nevertheless obtain multiple cellular HCV NS3/4A levels, varying concentrations of the HCV NS3/4A inhibitor Simeprevir are added to the setting. Therefore, an additional link between the intracellular HCV NS3/4A concentration and the administered protease inhibitor (Inh) is required:

$$NS34A={NS34A}_{0}\cdot\frac{K_{M,Inh}}{K_{M,Inh}+Inh}.$$

Combining the two equations allows us to describe remaining MAVS levels by

$$MAVS=\mathrm{MAVS}_{0}\cdot\frac{K_{M,NS34A}}{K_{M,NS34A}+{NS34A}_{0}\cdot\frac{K_{M,Inh}}{K_{M,Inh}+Inh}}.$$

HCV NS3/4A levels in absence of any inhibitor are set to 1 (${NS34A}_{0}=1$).

### 1.4.1 Quantifying the effect of viral antagonists on immune activation

We compare the simulated IFIT1 mRNA dynamics with experimentally observed ones in cells expressing CSFVNpro or DENVNS5. This is achieved by fitting the protein-specific K${}_{M}$ (for CSFVNpro, DENVNS5) to the experimental data. We set the viral protein concentration under the regulation of the stronger promotor to one and the viral protein concentrations under regulation of the weaker promotor are adjusted on the experimental data. The resulting model fits for CSFVNpro, DENVNS5 are shown below and the model fit for HCVNS34A in the main text.

|  |
| --- |

Figure 1.9: Upper panel: Model fit to experimental data of IFIT1 mRNA production in cells stably expressing viral proteins. The experimentally observed and fitted IFIT1 mRNA dynamics are shown for cells expressing viral proteins under the control of a strong promotor (green) a weaker promotor (red) and in control cells (black). Lower panel: Concentration-dependent alterations of the innate immune response by viral proteins. N${}_{pro}$ lowers the cellular IRF3 levels and NS5 the cellular STAT2 levels (here examplary shown for free cytoplasmic STAT2).

## 1.5 Simulation of RIG-I signalling in HepG2 cells

Cellular protein levels of our RIG-I model are updated based on protein copy numbers quantified by LC-MS/MS (see Table [1.13](#TabHepG2Initials)). Additionally, cell-specific properties, like the cytoplasmic and nuclear volume, are set to reported values from literature ($V_{\mathrm{cell}}=2.85e-12$ L , $V_{n}\approx6.9e-13$ L. $V_{n}$ adopted from HeLa cells (Bondarava 2008, Maul 1977). During the conducted simulations, the rate constants in our innate immune response model were not modified. We thereby assume that potential differences in innate immune signalling are due to cell-type specific expression of immune response components rather than differences in the kinetic rate constants.

Table 1.13: Changes in initial values of the coupled innate immune response model when adapting to HepG2 cells.

| Species | Protein concentrations [nM] | Compartment |
| --- | --- | --- |
| RNA | 1.95 | cytoplasm |
| RIGI | 6.76 | cytoplasm |
| MAVS | 0.302 | cytoplasm |
| IKK$\epsilon$ | 0 | cytoplasm |
| TBK1 | 0.015 | cytoplasm |
| IRF3 | 0.013 | cytoplasm |
| IKK$\beta$ | 7.62 | cytoplasm |
| iNF-$\kappa$B | 0 | cytoplasm |
| I$\kappa$B$\alpha$ | 0 | nucleus |
| NF$\kappa$B${}_{c}$ | 64.10 | cytoplasm |
| JAK | 26.06 | cytoplasm |
| TYK | 2.88 | cytoplasm |
| STAT1 | 215.01 | cytoplasm |
| STAT2 | 9.38 | cytoplasm |
| PIAS | 41.26 | nucleus |

##

## Profile likelihoods of the coupled immune response model

|  |
| --- |

Figure 1.10: Identifiability analysis of all parameters adjusted during model coupling.
