# Supplementary material for: High-resolution kinetic characterization of the RIG-I-signaling pathway and the antiviral response
Source: Life Sci Alliance. 2023 Aug 9;6(10):e202302059. doi: 10.26508/lsa.202302059 (PMC10412806; doi:10.26508/lsa.202302059)
Supplement: Supplementary file 2 [file LSA-2023-02059_TableS1.docx]

| Gene | Sequence (5’ – 3’) |
| --- | --- |
| IFNR DKO (double KO of IFNAR1  and IFNLR1) | gaccctagtgctcgtcgccg  caccggagtaccagatcatgccac |
| STAT2 | gtcgaatgtccacaggcagg |
