# Supplementary material for: High-resolution kinetic characterization of the RIG-I-signaling pathway and the antiviral response
Source: Life Sci Alliance. 2023 Aug 9;6(10):e202302059. doi: 10.26508/lsa.202302059 (PMC10412806; doi:10.26508/lsa.202302059)
Supplement: Supplementary file 3 [file LSA-2023-02059_TableS2.docx]

| Gene | Sequence (5’ – 3’) |
| --- | --- |
| GAPDH | tcggagtcaacggatttggt  ttcccgttctcagccttgac |
| IFIT1 | gaatagccagatctcagaggagc  ccatttgtactcatggttgctgt |
| IFNA | agccatctctgtcctccatgag  gatctcatgatttctgctctga |
| IFNB1 | cgccgcattgaccatcta  gacattagccaggaggttctc |
| IFNL1 (IL‑29) | ggtgactttggtgctaggct  tgagtgactcttccaaggcg |
| IFNL2/3 (IL‑28) | ctgccacatagcccagttca  agcgactcttctaaggcatct |
| TNFAIP3 | tcctcaggctttgtatttgagc  tgtgtatcggtgcatggtttta |
| MX1 | accattccaaggaggtgcag  tgcgatgtccacttcggaaa |
| RIG‑I | ccctggtttagggaggaaga  tcccaactttcaatggcttc |
| CCL5 | gctgtcatcctcattgctactg  tggtgtagaaatactccttgatgtg |
| ISG15 | acagccatgggctggga  ccttcagctctgacaccgac |
| ORF6 | atgtttcatctcgttgactttcagg  ttaatcaatctccattggttgctctt |
